# *Burkholderia cepacia* Complex Taxon K: Where to Split?

**DOI:** 10.3389/fmicb.2020.01594

**Published:** 2020-07-14

**Authors:** Eliza Depoorter, Evelien De Canck, Charlotte Peeters, Anneleen D. Wieme, Margo Cnockaert, James E. A. Zlosnik, John J. LiPuma, Tom Coenye, Peter Vandamme

**Affiliations:** ^1^Laboratory of Microbiology, Department of Biochemistry and Biotechnology, Faculty of Sciences, Ghent University, Ghent, Belgium; ^2^BCCM/LMG Bacteria Collection, Department of Biochemistry and Biotechnology, Faculty of Sciences, Ghent University, Ghent, Belgium; ^3^Division of Infectious Diseases, Department of Pediatrics, The University of British Columbia, Vancouver, BC, Canada; ^4^Department of Pediatrics, University of Michigan Medical School, Ann Arbor, MI, United States; ^5^Laboratory of Pharmaceutical Microbiology, Department of Pharmaceutical Analysis, Faculty of Pharmaceutical Sciences, Ghent University, Ghent, Belgium

**Keywords:** *Burkholderia*, novel species, genomic taxonomy, opportunistic pathogens, multilocus sequence typing

## Abstract

The objective of the present study was to provide an updated classification for *Burkholderia cepacia* complex (Bcc) taxon K isolates. A representative set of 39 taxon K isolates were analyzed through multilocus sequence typing (MLST) and phylogenomic analyses. MLST analysis revealed the presence of at least six clusters of sequence types (STs) within taxon K, two of which contain the type strains of *Burkholderia contaminans* (ST-102) and *Burkholderia lata* (ST-101), and four corresponding to the previously defined taxa Other Bcc groups C, G, H and M. This clustering was largely supported by a phylogenomic tree which revealed three main clades. Isolates of *B. contaminans* and of Other Bcc groups C, G, and H represented a first clade which generally shared average nucleotide identity (ANI) and average digital DNA-DNA hybridization (dDDH) values at or above the 95–96% ANI and 70% dDDH thresholds for species delineation. A second clade consisted of Other Bcc group M bacteria and of four *B. lata* isolates and was supported by average ANI and dDDH values of 97.2 and 76.1% within this clade and average ANI and dDDH values of 94.5 and 57.2% toward the remaining *B. lata* isolates (including the type strain), which represented a third clade. We therefore concluded that isolates known as Other Bcc groups C, G, and H should be classified as *B. contaminans*, and propose a novel species, *Burkholderia aenigmatica* sp. nov., to accommodate Other Bcc M and *B. lata* ST-98, ST-103, and ST-119 isolates. Optimized MALDI-TOF MS databases for the identification of clinical *Burkholderia* isolates may provide correct species-level identification for some of these bacteria but would identify most of them as *B. cepacia* complex. MLST facilitates species-level identification of many taxon K strains but some may require comparative genomics for accurate species-level assignment. Finally, the inclusion of Other Bcc groups C, G, and H into *B. contaminans* affects the phenotype of this species minimally and the proposal to classify Other Bcc group M and *B. lata* ST-98, ST-103, and ST-119 strains as a novel *Burkholderia* species is supported by a distinctive phenotype, i.e., growth at 42°C and lysine decarboxylase activity.

## Introduction

The *Burkholderia cepacia* complex (Bcc) is a group of closely related bacteria that occur naturally in a wide variety of ecological niches and that possess a remarkably versatile metabolism (Coenye et al., 2001; Parke and Gurian-Sherman, 2001; Depoorter et al., 2016; Kunakom and Eustáquio, 2019). Despite their agricultural potential, these organisms are perhaps best known as opportunistic pathogens, causing persistent infections in persons with cystic fibrosis (CF). The taxonomy of these bacteria is complex and continuously evolving. At present, Bcc consists of at least 22 validly named species (Peeters et al., 2013; De Smet et al., 2015; Bach et al., 2017; Martina et al., 2018). One additional species, i.e., “*Burkholderia paludis*” was effectively described but its name is awaiting validation (Ong et al., 2016).

One common group of Bcc bacteria, previously known as taxon K or group K, has presented a challenge in terms of classification (Vermis et al., 2002; Payne et al., 2005; Vanlaere et al., 2009). Taxon K now consists of two validly named species, *Burkholderia contaminans* and *Burkholderia lata*, and has been isolated from numerous human and environmental sources, including CF sputum, blood, cerebrospinal fluid, river water, sediment, soil, and various plants (Baldwin et al., 2005; Mahenthiralingam et al., 2006; Cesarini et al., 2009; da Silva et al., 2012). *B. contaminans* can be considered an emerging CF pathogen; it is the most common Bcc species in CF patients in Argentina (Cipolla et al., 2018) and it is increasingly isolated in CF centers in Spain (Medina-Pascual et al., 2015), the United Kingdom (Kenna et al., 2017), Portugal (Coutinho et al., 2015), and Ireland (Power et al., 2016). In addition, these organisms are frequently responsible for outbreaks of healthcare-associated infections due to contamination of pharmaceutical products such as nasal spray (CDC, 2004), dialysis water (Souza et al., 2004), moist washcloths (Martin et al., 2011), mouthwash (Zurita et al., 2014), and liquid docusate laxative (Glowicz et al., 2018).

A previous study that addressed the taxonomic position and structure of taxon K by means of *recA* gene sequence analysis and multilocus sequence typing (MLST) entailed the description of *B. contaminans* and *B. lata* (Vanlaere et al., 2009). However, the data presented indicated that the classification of taxon K bacteria was not completely resolved and would benefit from future study of a larger set of isolates. Today, many additional taxon K isolates have been collected through CF surveillance programs, environmental studies and outbreak investigations. In 2014, a study mining the Bcc PubMLST database using the 3% threshold value of average concatenated allele sequence divergence for species delineation (Vanlaere et al., 2009) revealed the presence of 16 putative novel Bcc species (Vandamme and Peeters, 2014), four of which (referred to as Other Bcc groups C, G, H, and M) grouped with *B. contaminans* and *B. lata* in a single taxon K clade that was supported by high bootstrap values (Vandamme and Peeters, 2014). The aim of the present study was to re-evaluate the taxonomy of taxon K bacteria. We performed a phylogenomic analysis to investigate established and potentially novel Bcc species within taxon K and we propose a revised classification of these common Bcc bacteria with considerable clinical relevance.

## Materials and Methods

### Bacterial Strains and Growth Conditions

Table 1 lists the sources of the 39 studied taxon K isolates. Isolates were grown aerobically on Tryptone Soya Agar (TSA, Oxoid CM0131) and incubated at 28°C. Cultures were preserved in MicroBank^TM^ vials at −80°C.

**TABLE 1:** Taxon K isolates included in the present study.

Isolate	Other strain designations	ST	Isolation source	Depositor
***Burkholderia contaminans***				
*B. contaminans* LMG 23253	VERONA 53	102	CF patient (Italy, 1997)	S. Campana
*B. contaminans* LMG 23361^T^	CCUG 55526^T^; J2956^T^	102	Sheep (Spain, 2000)	J. R. W. Govan
*B. contaminans* R-18175	IST 4120	96	CF patient (Portugal, 2002)	M. Cunha
*B. contaminans* R-18430	PC28	1401	Water (Brazil, 2001)	V. Magalhaes
*B. contaminans* R-18442	PC66	1403	Blood (Brazil, 2000)	V. Magalhaes
*B. contaminans* R-19218	Roma Ch (Sapienza) 16bc	345	CF patient (Italy)	G. Taccetti
*B. contaminans* R-37747	V02 33077	102	CF patient (Switzerland, 2006)	R. Zbinden
Other Bcc C AU33423	R-71068	1636	Non-CF sputum (United States, 2015)	Own isolate
Other Bcc C HI2714	R-71071	1637	Plant (United States)	Own isolate
Other Bcc C LMG 31807	BCC0517	1345	Plant (United States)	E. Mahenthiralingam
Other Bcc G AU15512	R-71058	1635	CF patient (United States, 2008)	Own isolate
Other Bcc G AU27893	R-71064	1638	CF patient (United States, 2013)	Own isolate
Other Bcc G LMG 31808	AU33803	535	CF patient (United States, 2016)	Own isolate
Other Bcc G HI4860	R-71073	1639	Docusate laxative (United States, 2016)	Own isolate
Other Bcc H AU6039	R-71057	1634	CF patient (United States, 2003)	Own isolate
Other Bcc H AU31652	R-71067	1640	CF patient (United States, 2015)	Own isolate
Other Bcc H HI2500	R-71070	1641	CF patient (United States)	Own isolate
Other Bcc H LMG 31806	BCC0132; CEP0657	934	CF patient (Canada)	D. P. Speert
Other Bcc H R-71171		863	CF patient (Belgium, 2017)	Own isolate
***Burkholderia lata***				
*B. lata* LMG 6860	ATCC 17771; NCIB9091	1404	Soil (Trinidad and Tobago, 1960)	NCIMB
*B. lata* LMG 6863	ATCC 17460; BCC0278; CEP0085; NCIB 9690	1415	River water (Trinidad and Tobago, 1965)	NCIMB
*B. lata* LMG 6992	ATCC 17769; CCUG 2857	1021	Soil (Trinidad and Tobago, 1960)	CCUG
*B. lata* LMG 6993	ATCC 17770; CCUG 2858; DSM 50180	1416	Soil (Trinidad and Tobago, 1960)	CCUG
*B. lata* LMG 14095	BCC0213; FC0450; 290/74	1520	CF patient (United Kingdom, 1974)	NCTC
*B. lata* LMG 22485^T^	383^T^; ATCC 17760^T^; DSM 23089^T^	101	Soil (Trinidad and Tobago, 1958)	T. Lessie
*B. lata* LMG 23254	R-11687; Saito 314	178	Seawater (Japan)	T. Saito
*B. lata* R-15816	323	1428	Plant (Brazil)	P. Hebbar
*B. lata* R-15945	275 SS	1521	Soil (Colombia)	P. Hebbar
*B. lata* R-18109	LSED4	456	Sediment (United States)	L. Chiarini
*B. lata* R-18110	LSED11	457	Sediment (United States)	L. Chiarini
*B. lata* R-18112	LWAT8	458	Sediment (United States)	L. Chiarini
*B. lata* R-39750	INPA42B	594	Soil (Brazil)	F. Souza Moreira
*B. lata* R-50215	PMP105	765	Soil (Argentina, 2011)	W. Draghi
***Burkholderia aenigmatica* sp. nov.**				
*B. lata* LMG 31805	CEP0088	98	Cerebrospinal fluid (United States)	D. P. Speert
*B. lata* R-9940	CEP1061; BCC0335	103	CF patient (Canada)	D. P. Speert
*B. lata* R-17378	HH 156.3	119	Car paint facility (Germany)	L. Eberl
*B. lata* R-18628	BCC0402; B5	119	Water (United States)	L. Leff
Other Bcc M AU17325	R-71061	1642	CF patient (United States, 2009)	Own isolate
Other Bcc M LMG 13014^T^	Div. 1680	333	Hand cream (Belgium)	S. Lauwers

*LMG, BCCM/LMG Bacteria Collection, Belgium; ATCC, American Type Culture Collection, United States; NCIMB, National Collection of Industrial, Food and Marine Bacteria, United Kingdom; CCUG, Culture Collection University of Gothenburg; NCTC, National Collection of Type Cultures, United Kingdom; CF, cystic fibrosis; ST, sequence type.*

### Genome Sequencing, Assembly and Annotation

Genomic DNA of 36 isolates (24 taxon K isolates and 12 Bcc type strains) was prepared from a 48-h culture at 28°C on TSA. Cells were harvested using a 10 μl inoculation loop and suspended in 300 μl of 4 M guanidine isothiocyanate solution (Fischer Scientific). DNA extraction was performed using the Maxwell^©^ 16 automated nucleic acid purification system using the Maxwell^©^ 16 Tissue DNA purification kit (Promega) according to the manufacturer’s instructions. Paired-end 150 bp libraries were sequenced on an Illumina HiSeq4000 sequencer (Oxford Genomics Centre – Wellcome Centre for Human Genetics, Oxford, United Kingdom). Quality control, assembly and annotation were performed as described previously (Peeters et al., 2019). Annotation was performed using Prokka v1.12 (Seemann, 2014) with a genus-specific database based on reference genomes from the *Burkholderia* Genome Database v8.1 (Winsor et al., 2008).

### Publicly Available Genomes

The publicly available whole-genome sequences of 12 taxon K isolates and 10 *Burkholderia* type strains were downloaded from the *Burkholderia* Genome Database and the NCBI database (Table 2). Publicly available raw Illumina reads from three taxon K isolates were downloaded from the European Nucleotide Archive (Table 2). These reads were assembled and annotated as described above.

**TABLE 2:** Genomic characteristics of *Burkholderia* genomes included in the present study.

Isolate	ST	Historical ST	BioProject	BioSample	Contigs	Size (bp)	N50 (bp)	CDS	%GC	References
***Burkholderia contaminans***										
*B. contaminans* LMG 23253	102	≡	PRJEB33447	SAMEA5780077	101	8651916	185096	7765	66.28	This study
*B. contaminans* LMG 23361^T^	102	≡	PRJNA203156	SAMN02402687	18	9263236	1108159	8038	65.89	Bloodworth et al., 2015
*B. contaminans* R-18175	96	≡	PRJEB33447	SAMEA5780078	54	8363746	341206	7461	66.51	This study
*B. contaminans* R-18430	1401	Incomplete	PRJEB33447	SAMEA5780079	128	8309019	148173	7490	66.31	This study
*B. contaminans* R-18442	1403	Incomplete	PRJEB33447	SAMEA5780080	68	8394843	308353	7508	66.37	This study
*B. contaminans* R-19218	345	≡	PRJEB33447	SAMEA5780081	82	7873816	189882	7126	66.18	This study
*B. contaminans* R-37747	102	≡	PRJEB33447	SAMEA5780082	163	8954430	106196	8115	66.07	This study
Other Bcc C AU33423	1636	≡	PRJNA391085	SAMN07257390	11	8328591	2082349	7349	66.57	
Other Bcc C HI2714	1637	≡	PRJNA391085	SAMN07257394	187	8937629	220096	7817	66.25	
Other Bcc C LMG 31807	1345	49	PRJEB33447	SAMEA5780083	92	8240073	195256	7358	66.63	This study
Other Bcc G AU15512	1635	≡	PRJNA391085	SAMN07257380	23	8210220	2136917	7198	66.88	
Other Bcc G AU27893	1638	≡	PRJNA391085	SAMN07257386	26	8583636	2738653	7637	66.53	
Other Bcc G LMG 31808	535	≡	PRJNA391085	SAMN07257392	28	8017538	1993215	7109	66.71	
Other Bcc G HI4860	1639	≡	PRJNA391085	SAMN07257396	67	8256473	560487	7345	66.61	
Other Bcc H AU6039	1634	≡	PRJNA391085	SAMN07257379	52	8490258	2107646	7454	66.68	
Other Bcc H AU31652	1640	≡	PRJNA391085	SAMN07257389	24	8240112	2091623	7230	66.84	
Other Bcc H HI2500	1641	≡	PRJNA391085	SAMN07257393	28	8265377	2158372	7307	66.89	
Other Bcc H LMG 31806	934	≡	PRJEB33447	SAMEA3752819	186	8406082	123770	7519	66.64	
Other Bcc H R-71171	863	≡	PRJEB33447	SAMEA5780084	47	8237582	287676	7295	66.81	This study
***Burkholderia lata***										
*B. lata* LMG 6860	1404	Incomplete	PRJEB33447	SAMEA5795691	144	8772945	142055	7780	66.36	This study
*B. lata* LMG 6863	1415	99	PRJEB33447	SAMEA5795695	173	8943181	103543	8001	66.19	This study
*B. lata* LMG 6992	1021	239	PRJEB33447	SAMEA3753241	46	8460613	377146	7526	66.58	
*B. lata* LMG 6993	1416	Incomplete	PRJEB33447	SAMEA5795696	70	8819830	240512	7934	66.35	This study
*B. lata* LMG 14095	1520	300	PRJEB33447	SAMEA5795693	98	7493810	146069	6800	66.29	This study
*B. lata* LMG 22485^T^	101	≡	PRJNA10695	SAMN02598262	3	8676277	3587082	7552	66.27	
*B. lata* LMG 23254	178	≡	PRJEB33447	SAMEA5795694	120	8876135	165805	8032	66.22	This study
*B. lata* R-15816	1428	Incomplete	PRJEB33447	SAMEA5795697	86	9163827	195689	8119	66.21	This study
*B. lata* R-15945	1521	422	PRJEB33447	SAMEA5795698	91	8869750	185209	7817	66.40	This study
*B. lata* R-18109	456	≡	PRJEB33447	SAMEA5795700	73	8958094	222909	7927	66.43	This study
*B. lata* R-18110	457	≡	PRJEB33447	SAMEA5795701	58	8968391	479277	7928	66.38	This study
*B. lata* R-18112	458	≡	PRJEB33447	SAMEA5795702	59	8554104	249949	7631	66.55	This study
*B. lata* R-39750	594	≡	PRJEB33447	SAMEA5795704	103	8816957	154064	7873	66.16	This study
*B. lata* R-50215	765	≡	PRJEB33447	SAMEA5795705	114	8840914	195256	7935	66.10	This study
***Burkholderia aenigmatica* sp. nov.**										
*B. lata* LMG 31805	98	≡	PRJEB33447	SAMEA3473589	91	9183824	232282	8441	65.60	
*B. lata* R-9940	103	≡	PRJEB33447	SAMEA5795706	90	7659857	188867	7034	66.35	This study
*B. lata* R-17378	119	342	PRJEB33447	SAMEA5795699	115	9328705	207151	8647	65.85	This study
*B. lata* R-18628	119	≡	PRJEB33447	SAMEA5795703	105	8639951	166930	7857	66.31	This study
Other Bcc M AU17325	1642	≡	PRJNA391085	SAMN07257383	104	9325364	355508	8410	65.51	
Other Bcc M LMG 13014^T^	333	≡	PRJEB33447	SAMEA5795692	158	8897342	98861	8336	65.95	This study
***Burkholderia* reference strains**										
*B. ambifaria* AMMD^T^	77	≡	PRJNA13490	SAMN02598309	4	7528567	2646969	6439	66.77	Johnson et al., 2015
*B. anthina* LMG 20980^T^	1346	86	PRJEB33447	SAMEA5780075	73	7608177	201942	6819	66.74	This study
*B. arboris* LMG 24066^T^	492	≡	PRJEB33447	SAMEA5780076	83	8271305	191329	7364	66.84	This study
*B. catarinensis* DNA89^T^	1327	≡	PRJNA338131	SAMN05521522	892	8118333	18250	6128	66.46	Bach et al., 2017
*B. cenocepacia* IIIA J2315^T^	28	≡	PRJNA339	SAMEA1705928	4	8055782	3217062	7114	66.90	Holden et al., 2009
*B. cepacia* ATCC 25416^T^	10	≡	PRJNA298860	SAMN04167160	4	8605945	3408190	7490	66.61	
*B. diffusa* LMG 24065^T^	164	≡	PRJEB33447	SAMEA5780085	87	7077396	201102	6309	66.26	This study
*B. dolosa* LMG 18943^T^	472	72	PRJEB33447	SAMEA5795690	165	6079078	81572	5248	66.97	This study
*B. latens* LMG 24064^T^	238	≡	PRJEB33447	SAMEA5795707	115	7075522	104925	6253	66.83	This study
*B. metallica* LMG 24068^T^	511	≡	PRJEB33447	SAMEA5795708	118	7532498	133502	6685	67.04	This study
*B. multivorans* ATCC BAA-247^T^	650	397	PRJNA264318	SAMN03140189	3	6322746	3428264	5543	67.24	Johnson et al., 2015
“*B. paludis*” LMG 30113^T^	1381	≡	PRJEB33447	SAMEA5795709	98	8627646	187105	7499	67.15	This study
*B. pseudomultivorans* LMG 26883^T^	536	≡	PRJEB33447	SAMEA5795710	150	7396511	93947	6712	66.99	This study
*B. puraquae* CAMPA 1040^T^	1065	≡	PRJNA381363	SAMN06675007	82	8097195	290133	7103	66.59	Leguizamon et al., 2017
*B. pyrrocinia* DSM 10685^T^	1631	41	PRJNA283474	SAMN03651233	4	7961346	3214757	6820	66.46	Kwak and Shin, 2015
*B. seminalis* LMG 24067^T^	473	≡	PRJEB33447	SAMEA5795711	197	7928753	86332	7151	67.07	This study
*B. stabilis* ATCC BAA-67^T^	51	50	PRJNA328254	SAMN05367054	3	8527947	3318880	7509	66.42	Bugrysheva et al., 2016
*B. stagnalis* LMG 28156^T^	787	≡	PRJEB33447	SAMEA5795712	242	7948011	61121	6980	67.18	This study
*B. territorii* LMG 28158^T^	791	≡	PRJEB33447	SAMEA5795713	101	6907990	129850	6033	66.65	This study
*B. thailandensis* E264^T^	488	≡	PRJNA10774	SAMN02604021	2	6723972	3809201	5632	67.63	Johnson et al., 2015
*B. ubonensis* LMG 20358^T^	299	≡	PRJEB33447	SAMEA5795714	153	7692957	84900	6745	67.24	This study
*B. vietnamiensis* LMG 10929^T^	379	65	PRJNA235223	SAMN03107475	4	6930496	2262093	5851	66.83	Johnson et al., 2015

*bp, base pairs; ST, sequence type; ≡, ST unchanged; CDS, coding sequences.*

### *recA* Gene Sequence Analysis

Partial *recA* gene sequences (663 bp) of the 61 *Burkholderia* isolates in the present study were extracted from the assembled whole-genome sequences using BLAST searches. The sequences were aligned based on their amino acid sequences using Muscle (Edgar, 2004) in MEGA7 (Kumar et al., 2016). A phylogenetic tree was constructed using the maximum likelihood method in RAxML version 8.2.12 (Stamatakis, 2014). Rapid bootstrapping and ML search were performed using the general time reversible model with CAT approximation (GTRCAT). Visualization and annotation of the phylogenetic tree was performed using iTOL (Letunic and Bork, 2019).

### MLST Analysis

Multilocus sequence typing allele sequences were extracted from assembled genomes through BLAST searches against the Bcc PubMLST website (Jolley and Maiden, 2010). Nucleotide sequences of each allele, allelic profiles and sequence types for all isolates in the present study are available on the Bcc PubMLST website^1^. For phylogenetic analysis, concatenated allele sequences were exported from the Bcc PubMLST database. Isolate metadata were processed using Rstudio and R version 3.4.4. A phylogenetic tree was constructed as described above.

### Phylogenomic Analyses

Pairwise digital DNA-DNA hybridization (dDDH) values and their confidence intervals were determined using the Genome-to-Genome Distance Calculator 2.1 (GGDC) (Meier-Kolthoff et al., 2013). Average nucleotide identity (ANI) values were calculated through OrthoANI using USEARCH (Lee et al., 2016; Yoon et al., 2017). Protein sequences were extracted from annotated genomes using the SeqIO utility of Biopython (Cock et al., 2009). BcgTree was used to extract the amino acid sequences of 107 essential bacterial single-copy core genes and to construct a phylogenomic tree through partitioned maximum-likelihood analysis (Ankenbrand and Keller, 2016). Visualization and annotation of the phylogenetic tree was performed using iTOL (Letunic and Bork, 2019).

### Matrix-Assisted Laser Desorption/Ionization Time-of-Flight Mass Spectrometry (MALDI-TOF MS) Analysis

All 39 taxon K isolates were grown on TSA (Oxoid CM0131) for 48 h at 28°C and were subcultivated twice prior to harvesting. Cell extracts were prepared as described previously (Wieme et al., 2014). For all isolates examined, both cell extracts (1 μl) and homogeneous cell smears of isolated colonies were transferred to the spot sites of a 96-well stainless steel target plate (Bruker Daltonics). Both cell smears and cell extracts were air-dried at room temperature. Each spot was overlaid with 1 μl of matrix solution (10 mg/ml α-cyano-4-hydroxycinnamic acid in acetonitrile:trifluoroacetic acid:MilliQ [50:2.5:47.5] water-solvent) and left to air-dry at room temperature. Cell smears were spotted in triplicate and cell extracts were spotted in duplicate to verify reproducibility. Prior to analysis, the mass spectrometer was externally calibrated using the Bacterial Test Standard (Bruker Daltonics) used for calibration. The target plate was measured twice automatically on a Bruker Microflex^TM^ LT/SH Smart platform (Bruker Daltonics). The spectra were obtained in linear, positive ion mode using FlexControl 3.4 software according to the manufacturer’s recommended settings (Bruker Daltonics).

The spectra of the cell smears and cell extracts were compared to those in the MALDI BioTyper *in vitro* diagnostics (MBT IVD) (DB-7712 MSP) library using the MBT Compass Explorer 4.1 software (Bruker Daltonics) according to the manufacturer’s default settings. The spectra were also compared with the combined spectra of the MBT IVD library and the LM-UGent in-house library, which consists of 298 main spectra (MSPs) representing 271 *Burkholderia* reference strains for which the identification has been verified through *recA* gene sequence analysis or MLST analysis, and which includes 17 taxon K strains (Supplementary Table 1). This in-house library was constructed by preparing a cell extract for each strain as described above, spotting each extract eight times on the target plate and measuring each spot four times to generate 32 spectra. FlexAnalysis 3.4 software (Bruker Daltonics) was used to assess the quality of the spectra generated. MBT Compass Explorer 4.1 software was used to generate MSPs based on a minimum of 24 spectra according to manufacturer’s instructions. Species-level identification was performed according to the Optimized Acceptance Criterium (OAC) (De Bel et al., 2011), meaning that the identification of the best matching spectrum was accepted in case of a log score ≥2.0 and when a 0.200 log difference was observed between the best matching spectrum and the highest-scoring spectrum of a different species.

### Biochemical Characterization and Cellular Fatty Acid Analysis

After a 24 h incubation period at 28°C on TSA (BD), a loopful of well-grown cells was harvested and fatty acid methyl esters were prepared, separated and identified using the Microbial Identification System (Microbial ID) as described previously (Vandamme et al., 1992). Biochemical characterization was performed as described previously (Henry et al., 2001).

## Results

### Whole-Genome Sequencing

To further characterize the taxonomic structure of *B. contaminans*, *B. lata* and related organisms, 61 isolates were selected for genome sequence analysis, including 39 taxon K isolates (24 newly sequenced, 12 publicly available whole-genome sequences and 3 publicly available sequencing read sets) and 22 *Burkholderia* reference strains (12 newly sequenced and 10 publicly available whole-genome sequences). The type strain of *Burkholderia thailandensis* was included as an outgroup. The whole-genome sequence of 36 isolates for which no genome sequence was publicly available was determined. The assembly of the Illumina HiSeq 150 bp paired-end reads resulted in assemblies with 46–242 contigs with N50 values ranging from 61 to 479 Kbp and a total genome size of 6.1–9.3 Mbp (Table 2). Both the raw Illumina reads and the annotated assemblies of these 36 genomes were submitted to the European Nucleotide Archive and are publicly available through the GenBank/EMBL/DDBJ accession numbers listed in Table 2. The genome sequences of the remaining isolates were publicly available (Table 2).

### MLST and *recA* Gene Sequence Analysis

**FIGURE 1:**
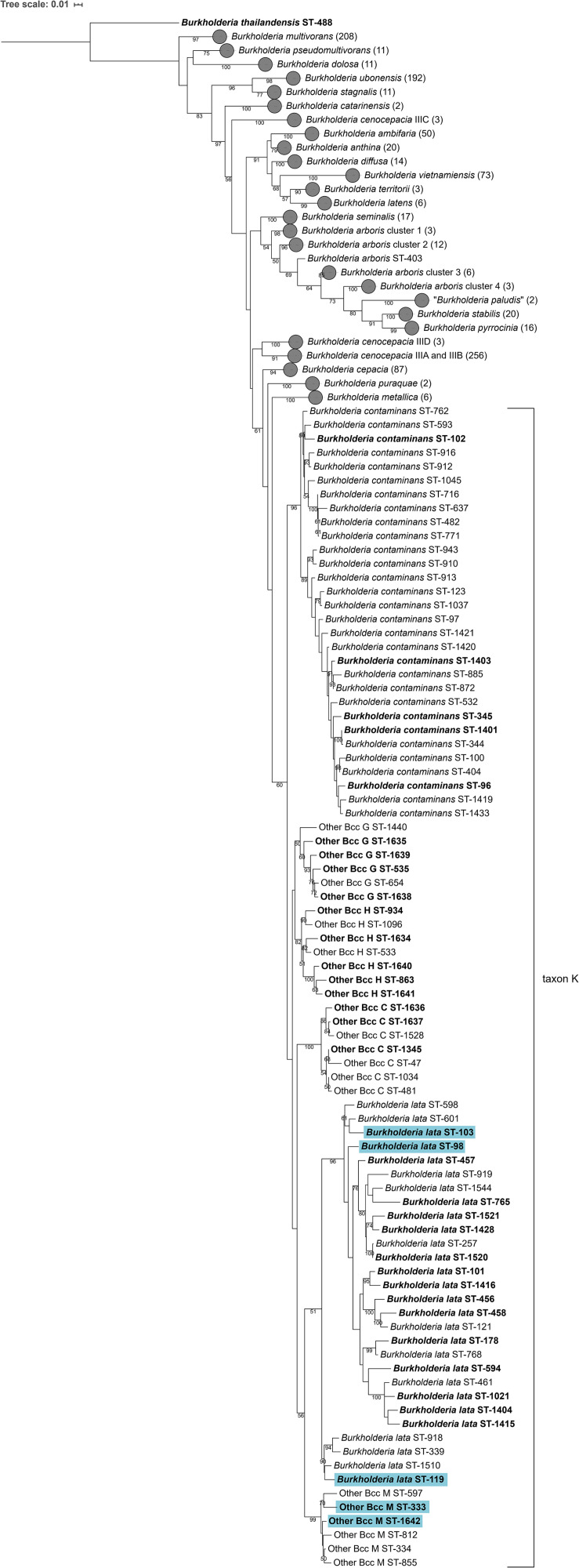
Phylogenetic tree based on the concatenated sequences (2760 bp) of seven housekeeping gene fragments of established Bcc species, taxon K strains and related Other Bcc isolates. Sequences [atpD (443 bp), gltB (400 bp), gyrB (454 bp), recA (393 bp), lepA (397 bp), phaC (385 bp), and trpB (301 bp)] corresponding to 1120 sequence types (STs) were downloaded from the Bcc PubMLST database (https://pubmlst.org/bcc/) (Jolley and Maiden, 2010) and aligned based on their amino acid sequences. Phylogeny was inferred using the Maximum Likelihood method and GTRCAT substitution model in RAxML. The percentage of replicate trees in which the associated taxa clustered together in the bootstrap test (1,000 replicates) are shown next to the branches if greater than 50%. The scale bar indicates the number of nucleotide substitutions per site. STs indicated in bold font were used for phylogenetic analyses and 39 taxon K STs indicated in bold were used for MALDI-TOF MS analyses. Gray circles indicate collapsed branches, the number of STs in such a collapsed branch is given after the species name between parentheses. STs that were reclassified into the novel species *Burkholderia aenigmatica* sp. nov. are marked in blue.

Although 16S rRNA gene sequence analysis is commonly used to identify the nearest neighbors of potentially novel taxa (Chun et al., 2018), the taxonomic resolution of this gene has proven insufficient to discriminate *Burkholderia* bacteria, in particular members of the Bcc (Vandamme and Dawyndt, 2011). Isolates were therefore assigned to the Other Bcc groups C, G, H, and M by means of MLST analysis as described previously (Vandamme and Peeters, 2014). Phylogenetic analysis of concatenated allele sequences of 1120 Bcc strains with unique STs present in the Bcc PubMLST database showed that taxon K strains represented a monophyletic clade (Figure 1). Within this clade, at least six clusters were observed. The type strains of *B. contaminans* LMG 23361^T^ and *B. lata* LMG 22485^T^ each resided in a stable cluster with bootstrap values of 96%. The Other Bcc group C, G, H, and M strain clusters were mostly supported by high bootstrap values (i.e., 100, 60, 82, and 99, respectively). A phylogenetic tree based on *recA* gene sequences is presented in Supplementary Figure 1.

### Phylogenomic Analyses

**FIGURE 2:**
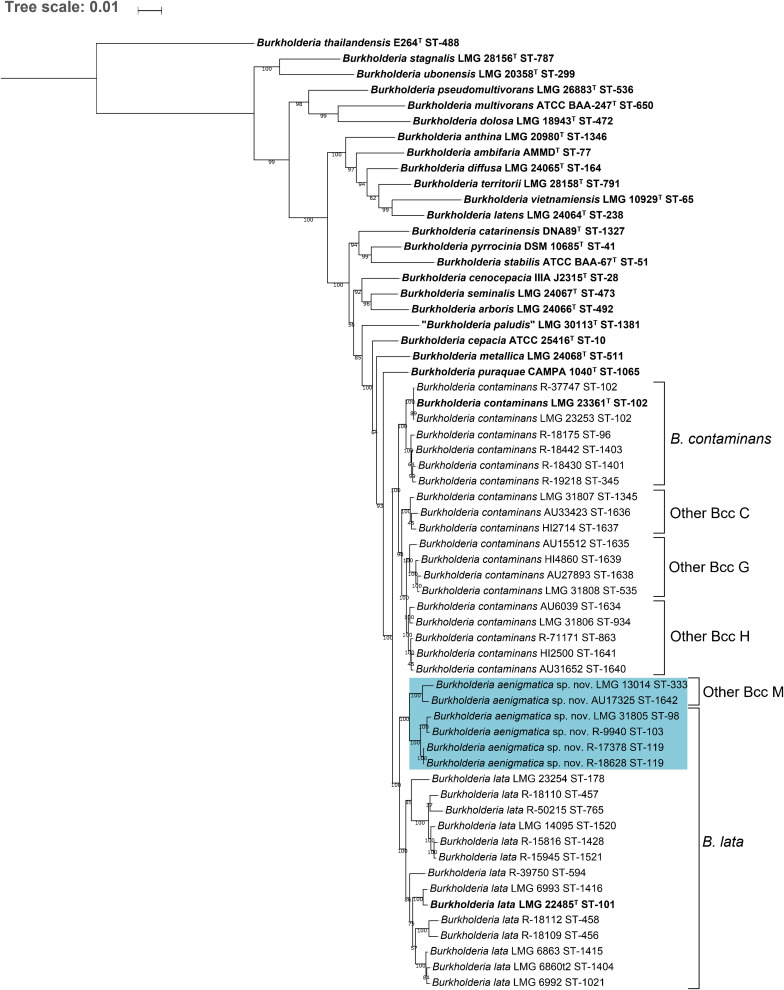
Whole-genome phylogeny of established Bcc species, taxon K strains and related Other Bcc group isolates. bcgTree was used to extract the amino acid sequences of 107 single-copy core genes from the genomes of 61 *Burkholderia* strains and to reconstruct their phylogeny through partitioned maximum-likelihood analysis (Ankenbrand and Keller, 2016). The percentage of replicate trees in which the associated taxa clustered together in the bootstrap test (1,000 replicates) are shown next to the branches if greater than 50%. The scale bar indicates the number of amino acid substitutions per site. Species names on the branches correspond to the taxonomic proposals in the present study, previous nomenclature is indicated next to braces on the right. Bold font indicates type strains of established Bcc species. Strains that were reclassified into the novel species *Burkholderia aenigmatica* sp. nov. are marked in blue.

The MLST dendrogram was used to select 39 taxon K isolates for genome sequence analyses (Figure 1, selected STs indicated in bold character type). The Maximum Likelihood phylogenetic tree resulting from the concatenated amino acid alignment of 107 single copy core genes was well-resolved and showed high bootstrap support on most branches (Figure 2). The *B. contaminans* and *B. lata* type strains were well-separated and grouped into distinct major clades. A first major clade included the *B. contaminans* type strain LMG 23361^T^ (ST-102) and consisted of four clusters (*B. contaminans* and Other Bcc groups C, G, and H), each supported by bootstrap values of 100%. A second major clade in the phylogenomic tree (Figure 2) included the *B. lata* type strain LMG 22485^T^ (ST-101) and consisted of four clusters of two to five isolates, supported by bootstrap values of 100% each, and two isolates with separate positions (i.e., R-39750 [ST-594] and LMG 23254 [ST-178]). *B. lata* isolates LMG 31805 (ST-98) and R-9940 (ST-103), which were present in a stable cluster (supported by a bootstrap value of 96%) with the *B. lata* type strain LMG 22485^T^ (ST-101) in the MLST tree (Figure 1), formed a distinct third clade together with the two ST-119 isolates R-17378 and R-18628 and the two Other Bcc M isolates in the phylogenomic tree. This third clade was supported by a bootstrap value of 100% (Figure 2).

The pairwise ANI and dDDH values of the 39 taxon K isolates and 22 *Burkholderia* reference strain genomes are presented in Supplementary Tables 2, 3 and average within-cluster and between-cluster ANI and dDDH values are presented in Table 3. Within the first major clade containing *B. contaminans* and Other Bcc groups C, G, and H, the average within-cluster ANI and dDDH values ranged from 97.7% ANI and 79.7% dDDH in Other Bcc G to 98.6% ANI and 87.4% dDDH in Other Bcc C (Table 3). The average between-cluster ANI and dDDH values ranged from 95.4% ANI and 62.4% dDDH between *B. contaminans* and Other Bcc C to 96.7% ANI and 71.2% dDDH between Other Bcc G and Other Bcc H (Table 3).

**TABLE 3:** Average within- and between-cluster ANI and dDDH values of seven clusters of taxon K isolates.

Group		Within-cluster ANI (%)			Within-cluster dDDH (%)			Between-cluster average ANI (%)						
		Average	Min	Max	Average	Min	Max	1	2	3	4	5	6	7
1	*B. contaminans* (*n* = 7)	98.2	97.3	99.9	84.7	76.9	99.8		95.4	95.5	95.5	94.5	94.0	93.8
2	Other Bcc C (*n* = 3)	98.6	98.4	98.8	87.4	85.2	89.5	62.4		95.8	96.2	94.8	94.2	94.1
3	Other Bcc G (*n* = 4)	97.7	96.9	98.8	79.7	73.2	89.4	63.1	64.7		96.7	94.6	94.1	94.0
4	Other Bcc H (*n* = 5)	98.3	97.8	99.2	85.1	80.4	93.8	63.1	68.0	71.2		94.8	94.3	94.2
5	*B. lata* (*n* = 14)	95.6	94.7	99.0	64.5	57.7	91.5	57.0	58.9	57.6	58.8		94.6	94.4
6	*B. lata* (*n* = 4)	98.9	98.6	99.9	90.5	87.4	99.7	54.3	55.8	55.0	55.8	57.5		95.8
7	Other Bcc M (*n* = 2)	97.6	NA	NA	78.2	NA	NA	53.6	55.1	54.4	55.1	56.4	65.0	

								1	2	3	4	5	6	7
								**Between-cluster average dDDH (%)**						

*All values are given in percent, n equals the number of isolates per cluster. ANI values are marked with gray background and are presented in the top right half of the matrix. dDDH values are presented in the bottom left half of the matrix. ANI, average nucleotide identity; dDDH, digital DNA-DNA hybridization; NA, not available.*

*Burkholderia lata* isolates belonging to the second major clade containing the *B. lata* type strain (Figure 2) appeared to yield a continuum of ANI and dDDH values in which several clusters of closely related strains could be distinguished. Strains (i) LMG 22485^T^ and LMG 6993, (ii) LMG 6860, LMG 6863 and LMG 6992, (iii) R-18109 and R-18112, and (iv) LMG 14095, R-18110, 15816, R-15945 and R-50215 revealed high pairwise ANI and dDDH each (i.e., values of at least 97.4 and 77.0%, respectively) (Supplementary Tables 2, 3). Values between strains of each of these four clusters and the two strains with unique positions (i.e., R-39750 and LMG 23254) ranged between 94.7 and 96.3% ANI, with an average of 95.2%; and between 57.9 and 68.5% dDDH, with an average of 60.9% (Supplementary Tables 2, 3).

Within the third major clade, the average within-cluster ANI and dDDH values ranged from 97.6% ANI and 78.2% dDDH in Other Bcc M to 98.9% ANI and 90.5% dDDH in the cluster containing *B. lata* isolates LMG 31805, R-9940, R-17378, and R-18628 (Table 3). Average between-cluster ANI and dDDH values of 95.8% ANI and 65.0% dDDH were observed between these two clusters (Table 3).

### MALDI-TOF MS Analysis

All 39 taxon K isolates included in the present study were analyzed and identified using MALDI-TOF MS in two ways. Analogous to the workflow in a routine clinical microbiology lab (Van Veen et al., 2010), isolates were analyzed by using the direct smear method and were identified by using the MBT IVD library. The resulting log scores of the best matching spectra are presented in Table 4 and ranged from 2.21–2.37 (*B. contaminans*), 2.09–2.27 (Other Bcc C), 2.15–2.24 (Other Bcc G), 2.24–2.32 (Other Bcc H), 2.15–2.52 (*B. lata*), and 2.21–2.24 (Other Bcc M). According to the optimized acceptance criteria for identification of Bcc bacteria (De Bel et al., 2011), only three taxon K strains could be identified to species level. *B. lata* strains LMG 22485^T^ and LMG 6993 were correctly identified, whereas *B. contaminans* LMG 23253 was misidentified as *B. cepacia*. The remaining isolates could not reliably be identified to species level and were identified as *B. cepacia* complex.

**TABLE 4:** Identification of taxon K isolates using MALDI-TOF MS.

	Cell smears identified using MBT IVD library			Cell extracts identified using MBT IVD library			Cell extracts identified using MBT IVD + in-house library		
Isolate	Consensus best match	Log score	Identification	Consensus best match	Log score	Identification	Consensus best match*	Log score	Identification
***Burkholderia contaminans***									
***B. contaminans* LMG 23253**	*B. cepacia* MB_7544_05	2.37	*B. cepacia*	*B. cepacia* MB_7544_05	2.45	*B. cepacia*	*B. contaminans* LMG 23361^T^	2.57	Bcc
***B. contaminans* LMG 23361^T^**	*B. cepacia* MB_7544_05	2.27	Bcc	*B. cepacia* MB_7544_05	2.41	Bcc	*B. contaminans* LMG 23361^T^	2.59	Bcc
*B. contaminans* R-18175	*B. cepacia* DSM 7288^T^	2.21	Bcc	*B. cepacia* MB_7544_05	2.36	Bcc	*B. contaminans* LMG 30304	2.72	*B. contaminans*
*B. contaminans* R-18430	*B. cepacia* DSM 7288^T^	2.28	Bcc	*B. cepacia* DSM 7288^T^	2.42	Bcc	*B. contaminans* LMG 30304	2.61	*B. contaminans*
*B. contaminans* R-18442	*B. cepacia* DSM 7288^T^	2.29	Bcc	No consensus	NA	Bcc	*B. contaminans* LMG 30304	2.71	*B. contaminans*
***B. contaminans* R-19218**	*B. cepacia* DSM 7288^T^	2.31	Bcc	No consensus	NA	Bcc	*B. contaminans* R-19218	2.74	*B. contaminans*
*B. contaminans* R-37747	*B. cepacia* MB_7544_05	2.34	Bcc	*B. cepacia* MB_7544_05	2.37	*B. cepacia*	*B. contaminans* LMG 23361^T^	2.47	Bcc
Other Bcc C AU33423	*B. cepacia* DSM 7288^T^	2.23	Bcc	*B. cepacia* DSM 7288^T^	2.27	Bcc	*B. contaminans* LMG 31807	2.49	Bcc
Other Bcc C HI2714	*B. cepacia* DSM 7288^T^	2.09	Bcc	*B. cepacia* DSM 50181	2.22	Bcc	*B. contaminans* LMG 31808	2.30	Bcc
**Other Bcc C LMG 31807**	*B. cepacia* DSM 7288^T^	2.27	Bcc	No consensus	NA	Bcc	*B. contaminans* LMG 31807	2.74	*B. contaminans*
Other Bcc G AU15512	*B. cepacia* MB_7544_05	2.15	Bcc	*B. cepacia* MB_7544_05	2.43	*B. cepacia*	*B. contaminans* LMG 23361^T^	2.52	Bcc
Other Bcc G AU27893	*B. cepacia* DSM 7288^T^	2.24	Bcc	*B. cepacia* MB_7544_05	2.37	Bcc	*B. contaminans* LMG 31807	2.55	Bcc
**Other Bcc G LMG 31808**	No consensus	NA	Bcc	*B. cepacia* DSM 7288^T^	2.37	Bcc	*B. contaminans* LMG 31808	2.71	*B. contaminans*
Other Bcc G HI4860	*B. cepacia* DSM 7288^T^	2.24	Bcc	*B. cepacia* DSM 7288^T^	2.36	Bcc	*B. contaminans* LMG 31807	2.55	Bcc
Other Bcc H AU6039	*B. cepacia* DSM 7288^T^	2.24	Bcc	*B. cepacia* MB_7544_05	2.31	Bcc	*B. contaminans* LMG 31806	2.57	Bcc
Other Bcc H AU31652	*B. lata* DSM 23089^T^	2.24	Bcc	*B. cepacia* MB_7544_05	2.36	Bcc	*B. contaminans* LMG 16227	2.58	Bcc
Other Bcc H HI2500	*B. cepacia* MB_7544_05	2.24	Bcc	*B. cepacia* MB_7544_05	2.40	Bcc	*B. contaminans* LMG 31808	2.53	Bcc
**Other Bcc H LMG 31806**	*B. cepacia* MB_7544_05	2.28	Bcc	*B. cepacia* MB_7544_05	2.46	*B. cepacia*	*B. contaminans* LMG 31806	2.67	*B. contaminans*
Other Bcc H R-71171	*B. cepacia* MB_7544_05	2.32	Bcc	*B. cepacia* MB_7544_05	2.38	Bcc	*B. contaminans* LMG 31806	2.49	Bcc
***Burkholderia lata***									
*B. lata* LMG 6860	*B. pyrrocinia* LMG 14191^T^	2.26	Bcc	*B. lata* DSM 23089^T^	2.38	Bcc	*B. lata* LMG 6992	2.49	Bcc
*B. lata* LMG 6863	*B. lata* DSM 23089^T^	2.28	Bcc	*B. lata* DSM 23089^T^	2.34	Bcc	*B. lata* LMG 6992	2.51	Bcc
***B. lata* LMG 6992**	*B. lata* DSM 23089^T^	2.29	Bcc	*B. pyrrocinia* LMG 14191^T^	2.31	Bcc	*B. lata* LMG 6992	2.50	Bcc
***B. lata* LMG 6993**	*B. lata* DSM 23089^T^	2.45	*B. lata*	*B. lata* DSM 23089^T^	2.60	*B. lata*	*B. lata* LMG 6993	2.62	Bcc
***B. lata* LMG 22485^T^**	*B. lata* DSM 23089^T^	2.52	*B. lata*	*B. lata* DSM 23089^T^	2.61	*B. lata*	*B. lata* LMG 22485^T^	2.63	*B. lata*
*B. lata* R-18110	*B. lata* DSM 23089^T^	2.31	Bcc	*B. lata* DSM 23089^T^	2.41	Bcc	*B. aenigmatica* sp. nov. R-18628	2.47	Bcc
*B. lata* R-50215	No consensus	NA	Bcc	*B. cepacia* DSM 7288^T^	2.21	Bcc	*B. lata* LMG 14095	2.34	Bcc
***B. lata* LMG 14095**	*B. cepacia* DSM 9241	2.15	Bcc	*B. pyrrocinia* LMG 14191^T^	2.17	Bcc	*B. lata* LMG 14095	2.55	*B. lata*
*B. lata* R-15816	No consensus	NA	Bcc	*B. metallica* DSM 23519^T^	2.20	Bcc	*B. lata* LMG 14095	2.31	Bcc
*B. lata* R-15945	*B. lata* DSM 23089^T^	2.15	Bcc	*B. metallica* DSM 23519^T^	2.25	Bcc	*B. lata* LMG 14095	2.31	Bcc
*B. lata* R-18109	*B. lata* DSM 23089^T^	2.21	Bcc	*B. stabilis* DSM 16586^T^	2.22	Bcc	*B. aenigmatica* sp. nov. LMG 31805	2.28	Bcc
*B. lata* R-18112	*B. lata* DSM 23089^T^	2.25	Bcc	*B. lata* DSM 23089^T^	2.35	Bcc	*B. aenigmatica* sp. nov. LMG 31805	2.40	Bcc
***B. lata* LMG 23254**	*B. lata* DSM 23089^T^	2.24	Bcc	*B. lata* DSM 23089^T^	2.37	Bcc	*B. lata* LMG 23254	2.64	Bcc
*B. lata* R-39750	*B. lata* DSM 23089^T^	2.39	Bcc	*B. metallica* DSM 23519^T^	2.27	Bcc	*B. lata* LMG 22485^T^	2.40	Bcc
***Burkholderia aenigmatica* sp. nov.**									
***B. lata* LMG 31805**	*B. lata* DSM 23089^T^	2.28	Bcc	*B. lata* DSM 23089^T^	2.32	Bcc	*B. aenigmatica* sp. nov. LMG 31805	2.62	Bcc
*B. lata* R-9940	*B. cepacia* DSM 7288^T^	2.19	Bcc	No consensus	NA	Bcc	*B. aenigmatica* sp. nov. R-18628	2.65	Bcc
*B. lataR-17378*	*B. cepacia* DSM 7288^T^	2.22	Bcc	*B. cepacia* DSM 9241	2.25	Bcc	*B. aenigmatica* sp. nov. R-18628	2.50	Bcc
***B. lata* R-18628**	*B. cepacia* DSM 50181	2.20	Bcc	*B. cepacia* DSM 9241	2.33	Bcc	*B. aenigmatica* sp. nov. R-18628	2.73	*B. aenigmatica* sp. nov.
Other Bcc M AU17325	*B. lata* DSM 50180	2.24	Bcc	*B. lata* DSM 50180	2.41	Bcc	*B. aenigmatica* sp. nov. LMG 13014^T^	2.49	Bcc
**Other Bcc M LMG 13014**	*B. cepacia* DSM 7288^T^	2.21	Bcc	*B. lata* DSM 50180	2.45	Bcc	*B. aenigmatica* sp. nov. LMG 13014^T^	2.68	*B. aenigmatica* sp. nov.

*Consensus best match was reported if the same best matching spectrum was observed for at least half of the replicate spectra (six for cell smears, four for cell extracts). The log score represents the average of log scores corresponding to the best matching spectrum. The identification of the best matching spectrum was accepted in case of a log score ≥2.0 and when a 0.200 log difference was observed between the best matching spectrum and the highest-scoring spectrum of a different species. MBT, MALDI BioTyper; IVD, *in vitro* diagnostics; ID, identification; Bcc, *Burkholderia cepacia* complex; NA, not available; ^∗^, species names in this column correspond to the taxonomic proposals in the present study. For strains indicated in bold type, MSPs were added to the in-house library to improve identification of taxon K bacteria.*

Isolates were also analyzed using a cell extract method and were identified by using the MBT IVD library. The resulting log scores from the best matching spectra are presented in Table 4 and ranged from 2.36–2.45 (*B. contaminans*), 2.22–2.27 (Other Bcc C), 2.36–2.43 (Other Bcc G), 2.31–2.46 (Other Bcc H), 2.17–2.61 (*B. lata*), and 2.41–2.45 (Other Bcc M). Six taxon K strains were considered identified to species level, only two of which were correctly identified as *B. lata* (LMG 22485^T^ and LMG 6993). Misidentification as *B. cepacia* was observed for two *B. contaminans* isolates (LMG 23253 and R-37747), one Other Bcc G strain (AU15512) and one Other Bcc H strain (LMG 31806). The remaining isolates could not reliably be identified to species level and were identified as *B. cepacia* complex.

To improve species-level identification, isolates were also analyzed using a cell extract method and were identified by using the spectra present in both the MBT IVD library and our in-house library of *Burkholderia* reference strains. The in-house library (Supplementary Table 1) included nine strains assigned below to *B. contaminans*, i.e., six *B. contaminans* strains (LMG 16227, LMG 23253, LMG 23361^T^, LMG 30304, R-18428 and R-19218), and one strain of each of the Other Bcc groups C (LMG 31807), G (LMG 31808) and H (LMG 31806); five strains belonging to the revised species *B. lata* (LMG 14095, LMG 22485^T^, LMG 23254, LMG 6992 and LMG 6993), and finally, three strains belonging to the proposed new taxon K species (LMG 13014^T^, LMG 31805 and R-18628) (see below). The resulting log scores from the best matching spectra are presented in Table 4 and ranged from 2.47–2.74 (*B. contaminans*), 2.30–2.74 (Other Bcc C), 2.52–2.71 (Other Bcc G), 2.49–2.67 (Other Bcc H), 2.28–2.73 (*B. lata*), and 2.49–2.68 (Other Bcc M). Eleven taxon K strains were correctly identified to species level, including four *B. contaminans* strains, one strain of each of the Other Bcc C, G, and H groups, two *B. lata* strains and two strains belonging to the proposed new species (Table 4). The remaining 28 isolates could not reliably be identified to the species level using the optimized acceptance criteria and were identified as *B. cepacia* complex. Nevertheless, the best matching spectra mostly represented isolates of the corresponding species.

### Biochemical Characterization and Cellular Fatty Acid Analysis

Biochemical characteristics and cellular fatty acid profiles were determined for seven representative strains: one strain from each of the Other Bcc groups C, G, H, and M and three additional *B. lata* isolates (LMG 23254, LMG 14095, and R-39750) The results are listed in Supplementary Table 4. Other Bcc M strain LMG 13014 could be distinguished from *B. lata* strains through growth at 42°C and lysine decarboxylase activity. The main fatty acid components are C_16:0_, C_17:0_ cyclo and C_18:1_ ω7c, in correspondence with the values reported previously for taxon K strains and Bcc bacteria in general (Vanlaere et al., 2009).

## Discussion

The Bcc PubMLST database serves as a framework to study the epidemiology and biodiversity of Bcc bacteria. To resolve the taxonomy of taxon K bacteria, a phylogenetic tree based on concatenated allele sequences of 1120 Bcc strains with unique STs was constructed. The resulting tree revealed the same four clusters of Other Bcc isolates, i.e., Other Bcc groups C, G, H, and M (names were adopted from Vandamme and Peeters, 2014) grouping with *B. contaminans* and *B. lata*, as reported earlier (Figure 1; Vandamme and Peeters, 2014). However, low bootstrap support for several nodes (Figure 1) and changing tree topologies upon analysis of different sets of STs from the PubMLST database (data not shown) prompted us to perform several whole-genome sequence-based comparisons. To this end, a total of 39 taxon K isolates (i.e., isolates belonging to *B. contaminans*, *B. lata* or Other Bcc groups C, G, H, and M) were selected based on the clustering result of the MLST tree (Figure 1) and their availability in our strain collections.

A phylogenomic tree was constructed based on the amino acid sequences of 107 conserved single-copy genes, extracted from the assembled genomes of 61 Bcc strains including 39 taxon K isolates. This tree generally supported the six clusters observed in the MLST tree but also revealed some discrepancies. A first cluster contained the type strain, several *B. contaminans* reference strains (Vanlaere et al., 2009) and recent isolates that were identified as *B. contaminans*, and therefore corresponded with the *recA*-I/MLST-I taxon reported by Vanlaere et al. (2009). The three remaining clusters corresponded with the other Bcc groups C, G, and H (Figure 2; Vandamme and Peeters, 2014). To investigate the degree of relatedness of these four clusters, pairwise OrthoANI and dDDH values were calculated from the assembled genomes (Table 3 and Supplementary Tables 2, 3). The average within-cluster ANI and dDDH values of these four clusters clearly exceeded the 95–96% ANI and 70% dDDH thresholds for species delineation (Wayne et al., 1987; Goris et al., 2007; Lee et al., 2016), whereas, the average between-cluster ANI and dDDH values were at or above these thresholds. Moreover, both ANI and dDDH values within the *B. contaminans* clade were clearly higher than those observed between the *B. contaminans* clade and other validly named Bcc species, or even between any two other validly named Bcc species.

A second major clade in the MLST tree (Figure 1) included the *B. lata* type strain LMG 22485^T^ (ST-101). Notably, the taxonomy of *B. lata* appeared unresolved in an earlier study (Vanlaere et al., 2009) with *B. lata* represented by two *recA* lineages (*recA*-II and *recA*-III) that were supported by low bootstrap values only. The same strains grouped in a single heterogeneous MLST lineage (MLST-II) and wet-lab DDH experiments revealed several values near the 70% DDH threshold for species delineation (Vanlaere et al., 2009). In the present study, the MLST tree revealed one major cluster of *B. lata* STs (Figure 1) and four additional STs that grouped with a low bootstrap support (51%) with the main *B. lata* cluster. Other Bcc M STs were well-separated from *B. lata* and all other taxon K strains and grouped with *B. lata* in the analysis shown in Figure 1, although this clustering was supported by a low bootstrap value only.

This same clustering of *B. lata* and Other Bcc M strains was not supported by the phylogenomic tree (Figure 2) where *B. lata* LMG 31805 (ST-98) and R-9940 (ST-103) no longer grouped with *B. lata* LMG 22485^T^ (ST-101) but clustered with *B. lata* R-17378 (ST-119) and R-18628 (ST-119), and with Other Bcc M STs and represented a third major clade. The latter clustering was supported by an average ANI value of 97.2% and an average dDDH value of 76.1% among the six strains examined, highlighting their close genomic relatedness (Supplementary Tables 2, 3). Values toward other *B. lata* strains ranged between 94.1 and 95.1% ANI and between 55.2 and 60.4% dDDH (Supplementary Tables 2, 3). Values toward other Bcc species ranged between 87.0 and 94.9 % ANI, and between 32.9 and 59.1% dDDH. The highest ANI and dDDH values were observed toward *Burkholderia puraquae* CAMPA1040^T^, which also appeared as a nearest neighbor of taxon K bacteria in the phylogenomic tree (Figure 2).

The remaining *B. lata* strains examined appeared to yield a continuum of ANI and dDDH values in which several clusters of closely related strains could be distinguished. Values toward other Bcc species ranged between 87.3 and 94.3% ANI, and between 33.1 and 56.1% dDDH. Again, the comparisons with *B. puraquae* CAMPA1040^T^ yielded the highest ANI and dDDH values (Supplementary Tables 2, 3).

Data of the present study therefore demonstrated that the taxon K bacteria examined represented three major genomic clades, and thus confirmed and extended earlier observations (Vanlaere et al., 2009). A first clade comprised *B. contaminans* and Other Bcc group C, G, and H strains; a second clade comprised the *B. lata* type strain and most other *B. lata* strains examined; and a third clade comprised the Other Bcc group M strains and some *B. lata* strains. While each of these major clades again comprised several clusters of closely related strains, especially the clade comprising the *B. lata* type strain appeared as a genomic continuum. Several genomic parameters have been proposed to replace the earlier wet-lab DDH for species delineation. ANI cutoffs of 94–95% (Konstantinidis and Tiedje, 2005) and 95–96% (Goris et al., 2007; Lee et al., 2016) and a dDDH cutoff of 70% (Wayne et al., 1987; Meier-Kolthoff et al., 2013) have been proposed and commonly allow straightforward interpretation of taxonomic data. Taxonomic proposals based on borderline values, such as many values obtained in the present study, are inevitably subjective. Small-scale comparisons of well-selected strains undoubtedly would allow to find some unique strains with a genomic divergence clearly beyond the threshold values and with a distinctive phenotype and would facilitate a straightforward description of several novel *Burkholderia* species (like for instance in reference Wallner et al., 2019). Yet, the complexity of the natural diversity as presented in this comprehensive dataset of taxon K bacteria argues against such practices.

*Burkholderia contaminans* and Other Bcc groups C, G, and H revealed ANI values that were near and above the species delineation threshold, while their dDDH values were near and below the species threshold (Supplementary Tables 2, 3). We previously introduced a 3% threshold value of average concatenated allele sequence divergence for species delineation within the *B. cepacia* complex (Vanlaere et al., 2009; Peeters et al., 2013). The inclusion of Other Bcc groups C, G, and H into *B. contaminans* would not affect this commonly used threshold as the average within-group divergence would become 2.2% [while 1.4% was reported earlier (Vanlaere et al., 2009)] and the average divergence between the nearest neighbor species of *B. contaminans* (including Other Bcc groups C, G, and H) would become 3.6% [while 3.5% was reported earlier (Vanlaere et al., 2009)].

The analysis of a considerable number of *B. lata* isolates in the present study further substantiated the complexity and the continued unresolved nature of the taxonomy of this species (Vanlaere et al., 2009). The discrepancies reported earlier between single and multilocus trees (Vanlaere et al., 2009) were extended into the discrepant topologies of the MLST and phylogenomic trees (Figures 1, 2). The ANI and dDDH analyses indicated that Other Bcc group M strains and *B. lata* ST-98, ST-103 and ST-119 strains should be classified as a novel Bcc species within taxon K. Although this was not apparent from the MLST tree (Figure 1), this was supported by the average concatenated allele divergence values: the average within-group divergence for this novel species was 2.8%; the average within-group divergence for *B. lata* was 2.4% [while 2.7% was reported earlier (Vanlaere et al., 2009)]. The average divergences between the three taxon K species were 3.1% between the novel species and *B. lata*, 3.3% between the novel species and *B. contaminans* (including Other Bcc groups C, G, and H) and 3.7% between *B. lata* and *B. contaminans* [including Other Bcc groups C, G, and H; while 3.5% was reported earlier (Vanlaere et al., 2009)].

The *recA* gene was introduced as a superior alternative to the 16S rRNA gene for discrimination of Bcc bacteria (Mahenthiralingam et al., 2000) and is still commonly used as a first-line identification method for putative Bcc bacteria. While *recA* gene sequence analysis remains a very useful tool for the identification of most Bcc species, the present study confirms that its use is problematic for the recognition of some Bcc species (Supplementary Figure 1).

In medical microbiology, MALDI-TOF MS commonly replaced traditional biochemical characterization as a first-line diagnostic instrument. We therefore analyzed the 39 taxon K isolates using the MALDI Biotyper system. By using the cell smear method and comparison of the resulting spectra with the MBT IVD database, we adhered to a routine workflow in a diagnostic laboratory. Of 39 isolates examined, only three were identified at the species level (all other isolates were identified as *B. cepacia* complex): *B. lata* isolates LMG 22485^T^ and LMG 6993 were correctly identified, whereas *B. contaminans* LMG 23253 was misidentified as *B. cepacia* (Table 4). The latter was not surprising since the MBT IVD database does not contain *B. contaminans* reference spectra. In addition, the entry for *B. lata* strain DSM 50180 (=LMG 6993) in the MBT IVD database is mislabeled as *B. cepacia*, which may contribute to misidentification of field isolates. In an effort to improve species-level identification, all 39 taxon K isolates were also analyzed using a cell extract method. Overall this resulted in somewhat higher log scores but it did not affect species-level identification positively. Indeed, while the same identification results were obtained for the *B. lata* strains LMG 22485^T^ and LMG 6993 (correctly identified), and *B. contaminans* LMG 23253 (misidentified), an additional three strains (*B. contaminans* R-37747, Other Bcc G AU15512 and Other Bcc H LMG 31806) were also misidentified as *B. cepacia* (Table 4). Finally, spectra derived from cell extracts were also identified by comparison with the spectra present in both the MBT IVD library and an in-house library of *Burkholderia* reference strains which included additional reference spectra of 17 taxon K bacteria (Supplementary Table 1) which were assigned to *B. contaminans*, *B. lata* and the novel taxon K Bcc species, as described below. This allowed correct species-level identification of seven *B. contaminans* strains, two *B. lata* strains and two strains belonging to the novel species; all remaining taxon K isolates were identified as *B. cepacia* complex. However, all but three strains examined had a strain corresponding to the correct species as their consensus best match (Table 4) confirming that the use of optimized MALDI-TOF MS databases can improve species-level identification of clinical Bcc bacteria (Degand et al., 2008; Fernández-Olmos et al., 2012; Alby et al., 2013).

## Conclusion

*Burkholderia* bacteria, and Bcc bacteria in particular, attracted a lot of interest because of their biocontrol and other biotechnological properties, but even more so as life-threatening pathogens in persons with CF (Mahenthiralingam et al., 2008; LiPuma, 2010). Among Bcc bacteria, taxon K became well-known as a notorious contaminant of pharmaceutical products (Torbeck et al., 2011; Cunningham-Oakes et al., 2020; Tavares et al., 2020). The present study was initiated to clarify the unresolved taxonomy of taxon K bacteria but revealed many borderline values which prevented a straightforward interpretation of data. While data from the present study can be used to argue that each of the clusters of taxon K strains with ANI and dDDH values well above the commonly applied cutoff levels corresponds with a distinct species or subspecies, such a classification would not bear a practical purpose and would render communication exceedingly complex. The classification of Other Bcc group C, G, and H bacteria as *B. contaminans*, and the proposal of a separate species status for Other Bcc group M bacteria and some strains now classified as *B. lata* acknowledges the genomic divergence between these three main clades but does not yield a simple identification scheme. The use of optimized MALDI-TOF MS databases for the identification of clinical *Burkholderia* isolates (Table 4) may provide accurate species-level identification for some of these bacteria; others will be identified as Bcc only. The public deposit of a reference strain of each of these Other Bcc groups into the BCCM/LMG Bacteria Collection^2^ (Table 1) will allow improvement of MALDI-TOF MS databases. Although MLST facilitates species-level identification of many taxon K strains, comparative genomics may be required to identify some recalcitrant strains. Finally, although phenotypic tests are rarely used today for species-level identification of Bcc bacteria (Vandamme and Dawyndt, 2011), the inclusion of Other Bcc groups C, G, and H into *B. contaminans* affects the phenotype of this species minimally (Supplementary Table 4) and the proposal of a novel species status for Other Bcc group M and *B. lata* ST-98, ST-103, and ST-119 strains is supported by their distinctive phenotype [i.e., growth at 42°C and lysine decarboxylase activity distinguish the novel species from *B. lata* (Supplementary Table 4)].

### Description of *Burkholderia aenigmatica* sp. nov.

*Burkholderia aenigmatica* sp. nov. (ae.nig.ma’ti.ca. L. fem. adj. aenigmatica, enigmatic, referring to its unusual and complex taxonomy).

*Burkholderia aenigmatica* cells are Gram-negative, non-sporulating, straight rods. All strains grow on *B. cepacia* selective agar and MacConkey agar. Oxidase, ornithine decarboxylase and gelatinase activity is present. No lysine decarboxylase, β-galactosidase, arginine dihydrolase, urease, esculin hydrolase or nitrate reductase activity. Growth is observed at 30 and 42°C. Assimilation of glucose, L-arabinose, D-mannose, D-mannitol, *N*-acetyl-glucosamine, D-maltose, D-gluconate, caprate, adipate, L-malate, citrate, and phenylacetate. Acidification of glucose and lactose; not of maltose, xylose, sucrose and adonitol. The G+C content is between 65.5 and 66.3 mol%. The following fatty acids are present in all strains: C_14:0_, C_16:0_, cyclo C_17:0_, C_16:0_ 2-OH, C_16:0_ 3-OH, C_16:1_ 2-OH, C_18:1_ 2-OH, C_18:1_ ω7c, cyclo C_19:0_ ω8c, summed feature 2 (comprising C_14:0_ 3-OH, iso C_16:1_ I, an unidentified fatty acid with equivalent chain-length of 10.928 or C_12:0_ ALDE or any combinations of these fatty acids), summed feature 3 (comprising C_16:1_ ω7c or iso C_15_ 2-OH or both) and summed feature 7 (comprising C_18:1_ ω7c, C_18:1_ ω9t, C_18:1_ ω12t or any combination of these fatty acids). *B. aenigmatica* strains have been isolated from the environment and the respiratory tract of CF patients.

The type strain is LMG 13014^T^. Phenotypic characteristics of the type strain are the same as described above for the species. Its G+C content is 66.0 mol% and its 16S rRNA, *recA* and whole-genome sequences are publicly available through the accession numbers LR760817, LR761314, and CABVQC000000000, respectively. Strain LMG 13014^T^ was isolated as a contaminant of hand cream in Belgium in 1991 (Vandamme et al., 1997).

### Emended Description of *Burkholderia contaminans* Vanlaere et al., 2009

The description of *B. contaminans* is the one given by Vanlaere et al. (2009) with the following modifications: β-galactosidase activity and acidification of lactose and D-xylose are strain-dependent, rather than consistently present. *B. contaminans* includes the taxa known as Other Bcc groups C, G, and H. The G+C content of the type strain is 65.9 % and its whole-genome sequence is publicly available through the accession number GCA_000987075.1.

### Emended Description of *Burkholderia lata* Vanlaere et al., 2009

The description of *B. lata* is the one given by Vanlaere et al. (2009) with the following modifications: acidification of maltose, lactose and D-xylose are strain-dependent, rather than consistently present. The G+C content of the type strain is 66.3% and its whole-genome sequence is publicly available through the accession number GCA_000012945.1.

## Data Availability Statement

The datasets presented in this study can be found in online repositories. The names of the repository/repositories and accession number(s) can be found below: https://www.ncbi.nlm.nih.gov/bioproject/PRJEB33447.

## Author Contributions

ED and PV conceived the study and wrote the manuscript. ED, PV, and TC proofread the manuscript. ED and EDC performed all experiments except the biochemical characterization. MC and JZ performed the biochemical characterization. ED and CP carried out the genomic data analyses. ED and AW analyzed the MALDI-TOF MS data. JL isolated the strains. All authors read and approved the final manuscript.

## Conflict of Interest

The authors declare that the research was conducted in the absence of any commercial or financial relationships that could be construed as a potential conflict of interest.

## Footnotes

1 https://pubmlst.org/bcc/

2 http://bccm.belspo.be/

## Abbreviations

Bcc: *Burkholderia cepacia* complex
MLST: multilocus sequence typing
ST: sequence type
ANI: average nucleotide identity
dDDH: digital DNA-DNA hybridization
MALDI-TOF MS: matrix-assisted laser desorption/ionization time-of-flight mass spectrometry
CF: cystic fibrosis
GGDC: Genome-to-Genome Distance Calculator
MBT IVD: MALDI BioTyper *in vitro* diagnostics
MSP: main spectrum.
