# Supplementary material for: Burkholderia cepacia Complex Taxon K: Where to Split?
Source: Front Microbiol. 2020 Jul 14;11:1594. doi: 10.3389/fmicb.2020.01594 (PMC7372133; doi:10.3389/fmicb.2020.01594)
Supplement: Supplementary file 5 [file Image_1.pdf]

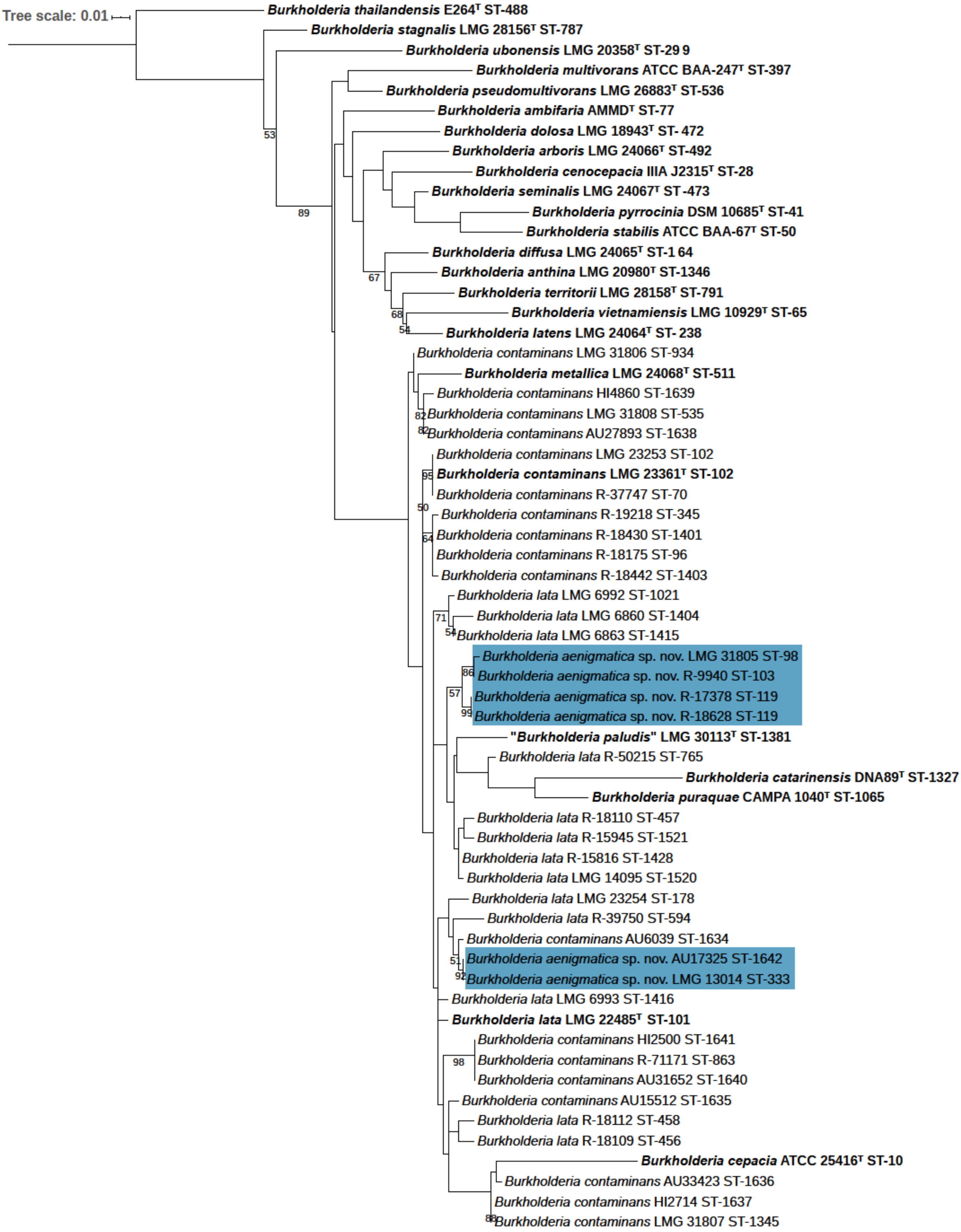

Tree scale: 0.01
Burkholderia thailandensis E264T ST-488
Burkholderia stagnalis LMG 28156T ST-787
Burkholderia ubonensis LMG 20358T ST-29 9
Burkholderia multivorans ATCC BAA-247T ST-397
Burkholderia pseudomultivorans LMG 26883T ST-536
Burkholderia ambifaria AMMDT ST-77
Burkholderia dolosa LMG 18943T ST- 472
Burkholderia arboris LMG 24066T ST-492
Burkholderia cenocepacia IIIA J2315T ST-28
Burkholderia seminalis LMG 24067T ST -473
Burkholderia pyrrocinia DSM 10685T ST-41
Burkholderia stabilis ATCC BAA-67T ST-50
Burkholderia diffusa LMG 24065T ST-1 64
Burkholderia anthina LMG 20980T ST-1346
Burkholderia territorii LMG 28158T ST-791
Burkholderia vietnamiensis LMG 10929T ST-65
Burkholderia latens LMG 24064T ST- 238
Burkholderia contaminans LMG 31806 ST-934
Burkholderia metallica LMG 24068T ST-511
Burkholderia contaminans HI4860 ST-1639
Burkholderia contaminans LMG 31808 ST-535
Burkholderia contaminans AU27893 ST-1638
Burkholderia contaminans LMG 23253 ST-102
Burkholderia contaminans LMG 23361T ST-102
Burkholderia contaminans R-37747 ST-70
Burkholderia contaminans R-19218 ST-345
Burkholderia contaminans R-18430 ST-1401
Burkholderia contaminans R-18175 ST-96
Burkholderia contaminans R-18442 ST-1403
Burkholderia lata LMG 6992 ST-1021
Burkholderia lata LMG 6860 ST-1404
Burkholderia lata LMG 6863 ST-1415
Burkholderia aenigmatica sp. nov. LMG 31805 ST-98
Burkholderia aenigmatica sp. nov. R-9940 ST-103
Burkholderia aenigmatica sp. nov. R-17378 ST-119
Burkholderia aenigmatica sp. nov. R-18628 ST-119
"Burkholderia paludis" LMG 30113T ST-1381
Burkholderia lata R-50215 ST-765
Burkholderia catarinensis DNA89T ST-1327
Burkholderia puraquae CAMPA 1040T ST-1065
Burkholderia lata R-18110 ST-457
Burkholderia lata R-15945 ST-1521
Burkholderia lata R-15816 ST-1428
Burkholderia lata LMG 14095 ST-1520
Burkholderia lata LMG 23254 ST-178
Burkholderia lata R-39750 ST-594
Burkholderia contaminans AU6039 ST-1634
Burkholderia aenigmatica sp. nov. AU17325 ST-1642
Burkholderia aenigmatica sp. nov. LMG 13014 ST-333
Burkholderia lata LMG 6993 ST-1416
Burkholderia lata LMG 22485T ST-101
Burkholderia contaminans HI2500 ST-1641
Burkholderia contaminans R-71171 ST-863
Burkholderia contaminans AU31652 ST-1640
Burkholderia contaminans AU15512 ST-1635
Burkholderia lata R-18112 ST-458
Burkholderia lata R-18109 ST-456
Burkholderia cepacia ATCC 25416T ST-10
Burkholderia contaminans AU33423 ST-1636
Burkholderia contaminans HI2714 ST-1637
Burkholderia contaminans LMG 31807 ST-1345
53
89
67
68
54
82
82
95
50
64
71
54
86
57
99
51
92
98
88

Supplementary Figure 1. Phylogenetic tree based on partial *recA* sequences of the 61 *Burkholderia* strains examined in the present study. Sequences (663 bp) were aligned based on their amino acid sequences and phylogeny was inferred using the Maximum Likelihood method and GTRCAT substitution model in RAxML. The percentage of replicate trees in which the associated taxa clustered together in the bootstrap test (1000 replicates) are shown next to the branches if greater than 50%. *Burkholderia thailandensis* E264^T was used as outgroup. The scale bar indicates the number of substitutions per site. Type strains of validly named Bcc species are shown in bold character type. Strains that were reclassified into the novel species *Burkholderia aenigmatica* sp. nov. are marked in blue.
